# Role of the microbiome and its metabolites in ankylosing spondylitis

**DOI:** 10.3389/fimmu.2022.1010572

**Published:** 2022-10-13

**Authors:** Zi-Yi Song, Duo Yuan, Sheng-Xiao Zhang

**Affiliations:** ^1^ Department of Rheumatology, The Second Hospital of Shanxi Medical University, Taiyuan, China; ^2^ Key Laboratory of Cellular Physiology at Shanxi Medical University, Ministry of Education, Taiyuan, China; ^3^ Shanxi Provincial Key Laboratory of Rheumatism Immune Microecology, The Second Hospital of Shanxi Medical University, Taiyuan, China; ^4^ Department of Gynecology and Obstetrics, The Second Hospital of Shanxi Medical University, Taiyuan, China

**Keywords:** ankylosing spondylitis, HLA-B27, microbiota, microbiome, microbial metabolites

## Abstract

Ankylosing spondylitis (AS), a chronic condition that commonly influences the spine and sacroiliac joints, usually progresses to stiffness and progressive functional limitation. Its fundamental etiology and pathogenesis are likely multifactorial and remain elusive. As environmental factors, gut microbiota performs critical functions in the pathogenesis of AS through various mechanisms, including interacting with genes, enhancing intestinal permeability, activating the gut mucosa immune system, and affecting the intestinal microbiota metabolites. This review provides an overview of recent advances in investigating gut microbiota in AS pathogenesis and discusses potential methods for future therapeutic intervention.

## 1 Introduction

Ankylosing spondylitis (AS), a prototype of spondyloarthritis (SpA), affects the fibrous and synovial joints of the sacroiliac and spinal joints, with a prevalence estimated between 0.07% and 0.31% in the world population (1). Its sphere may incorporate extra-articular features, including anterior uveitis and inflammatory bowel diseases (IBD) such as Crohn’s disease (CD) and ulcerative colitis (UC). The etiological mechanism of AS is complex, developing *via* the convergence of genetic, microbial, environmental, and immunological factors (2–4). Notwithstanding, emerging evidence suggest the role of environmental factors such as the intestinal microbiome.

The microorganisms colonized in the human gastrointestinal tract mucosa are collectively called the gut microbiota or gut microbiome, which mainly includes symbiotic bacteria, fungi, and viruses. The gut commensal bacteria, also known as the “microbial organ”, are the most significant and complex component of the host immune system. This microbial organ is crucial for the mucosal immune system’s initiation, upkeep, and operation. In the early stages of the disease, the diversity, structure, and function of the gut microbiota differ from healthy individuals. The microbiota dysbiosis affects the immune system locally and throughout the body, thus predisposing humans to several pathologies, including AS, IBD, reactive arthritis, etc. (5–7). The objective of our review is to present the current comprehension of the role of the gut microbiome and its metabolites in the pathogenesis of AS and to further explore potential therapeutic avenues targeting the gut microbiome for the clinical management of AS.

## 2 Gut microbiota and AS

Recent studies have shown that the occurrence and development of AS are related to changes in the diversity and composition of the gut microbiome (**Table 1**). Zhou et al. (5) found that *Bacteroides coprophilus, Parabacteroides distasonis, Eubacterium siraeum, Acidaminococcus fermentans, Prevotella copri* were enriched in AS, while *Enterococcus faecium E980* and *TX0133a01* were reduced by metagenomic shotgun sequencing. Liu et al. (11) performed 16S rRNA gene sequencing on stool samples from AS patients and healthy controls (HCs) and found that compared with HCs, relative abundance of *Bacteroidetes* was decreased in AS cases, but *Firmicutes* and *Verrucobacterium* were increased. Morever, specific gut bacteria were associated with disease activity of AS patients. Costello et al. (17) found increased abundances of *Lachnospiriaceae*, *Ruminococcus*, *Rikenellaceae*, *Porphyromonadaceae* and *Bacteroidaceae*, but decreased abundance of *Veillonellaceae* and *Prevotellaceae* in the terminal ileum of AS patients. These findings suggest that some AS-rich species may be triggers of autoimmunity. The *Firmicutes/Bacteroidetes* (F/B) ratio has been widely associated with maintaining normal intestinal homeostasis, and an increased abundance of specific *Firmicutes* species causes AS (11, 14). In addition, the researchers also observed changes in metabolites outside and inside the gut in AS patients (**Table 2**). The changes of intestinal metabolites are closely related to intestinal microbial metabolism, and the changes of extra-intestinal metabolites may also be partly related to the transfer of intestinal microbial metabolites.

**Table 1:** Alterations in gut microbiota in stool samples from AS patients compared to HCs.

Research object	Technology employed	Alterations in diversity		Increased Taxa	Decreased Taxa	Ref.
		α-diversity	β-diversity			
28 AS patients and 32 HCs in Romania	qPCR	Decreased but without any statistical value (gave no exact details)		*Bifidobacterium, Lactobacillus, Escherichia coli*	*Bacteroides, Clostridium coccoides (XIVa), Clostridium leptum (IV), Faecalibacterium prausnitzii*	(8)
85 AS patients and 62 HCs in China	Metagenomic shotgun sequencing	Decreased (Shannon index)	Difference (PCoA)	*Bacteroides coprophilus DSM_18228, Parabacteroides distasonis ATCC_8503, Eubacterium siraeum V10Sc8a, Acidaminococcus fermentans VR4_DSM_20731-mgs0807, Prevotella copri CB7_DSM_18205*	*Enterococcus faecium E980, TX0133a01*	(5)
11 AS patients and 37 HCs in China	Metagenomic shotgun sequencing	No significant difference (Shannon index)	ND	*Flavonifractor plautii, Oscillibacter, Parabacteroides distasonis, Bacteroides nordii*	*Collinsella aerofaciens*	(9)
20 AS patients and 20 HCs (ASns: 11; ASs: 9; HCns: 10; HCss: 10) in China	16S rRNA gene sequencing	ASns0 and HCns: ASns0 > HCns (Shannon, ACE, and Chao1 indices), no significant difference (Simpson index); ASs0 and HCss, HCns and HCss: no significant difference	ASns0 > ASs0, HCns > HCss, HCns > ASns0, ASs0 > HCss (Bray_curtis analysis)	*g_Comamonas* and *g_Desulfovibrio* (ASns > ASs); *g_Actinomyces*, *g_Collinsella*, *g_Lachnospiraceae_UCG-008*, and *g_Paraprevotella* (ASs > ASns).		(10)
10 AS patients and 12 HCs in China	16S rRNA gene sequencing	No significant difference (Shannon, Simpson, Chao1, and ACE indices)	ND	*p_Firmicutes, p_Verrucomicrobia, g_Bacteroides*	*p_Bacteroidetes, g_Ruminococcus, g_Helicobacter, g_Parasutterella*	(11)
20 AS patients and 19 HCs in China	16S rRNA gene sequencing	No significant difference (Shannon and Simpson indices), increased (Chao1 and ACE indices)	ASs0 < HCs (PCA with unweighted binary-Jaccard method)	*f_Prevotellaceae, f_Actinomycetaceae, g_Dialister, g_Escherichia-Shigella, g_Klebsiella*	*f_Lachnospiraceae, g_Bacteroides, g_Parasutterella, g_Bifidobacterium*	(12)
41 AS patients (20 axAS and 21 pAS) and 19 HCs in China	16S rRNA gene sequencing	No significant difference (Shannon and Simpson indices)	Difference (PCoA with unweighted UniFrac)	AS: *Prevotella_9, Dialister, Comamonas, Collinsella, Streptococcus, AlloPrevotella, Prevotella_2;* pAS: *Comamonas, Streptococcus and Collinsella;* axAS: *Prevotella_2*	*Eubacterium_ruminantium_group, Ruminococcus_gnavus_group, Lachnospira, Bacteroides*	(13)
22 AS patients and 16 HCs in China	16S rRNA gene- and ITS2-based DNA sequencing	Bacteria: increased (Observed species, Shannon and Simpson indices). Fungi: decreased (observed species and Shannon index)	Bacteria: no significant difference. Fungi: difference (PCoA)	*Bacteria: p_Proteobacteria, g_Escherichia-Shigella, g_Veillonella, g_Faecalibacterium, g_Eubacterium rectale group, g_Streptococcus, g_Lachnospiraceae NK4A136 group. Fungi: p_Ascomycota, c_Dothideomycetes)*	*Bacteria: p_Bacteroidetes, g_Prevotella strain 9, g_Megamonas, g_Fusobacterium. Fungi: p_Basidiomycota, o_Agaricales*	(14)
150 AS patients and 17 HCs in Sweden	GA-map™ Dysbiosis Test	ND	Difference (PCA)	*Proteobacteria, Enterobacteriaceae, Bacilli, Streptococcus species, Actinobacteria*	*Bacteroides, Lachnospiraceae*	(15)
97 AS patients and 114 HCs in China	Metagenomic shotgun sequencing	Decreased at species level and no difference at genus level (Shannon and Simpson indices)	ND	*Prevotellaceae (Prevotella melaninogenica, Prevotella copri, Prevotella* sp. *C561), Actinobacteria (Neisseria, Bifidobacterium, Collinsella, Rothia, and Actinomyces)*	*Bacteroides* spp.*, Enterobacter, Citrobacter, Fusobacteria, Verrucomicrobia, Enterobacteriaceae (Fusobacterium), Lachnospiraceae bacterium*	(16)
9 AS patients and 9 HCs in Australia	16S rRNA gene sequencing	Increased (Observed species)	Difference (PCoA)	*Lachnospiraceae, Ruminococcaceae, Rikenellaceae, Porphyromonadaceae, Bacteroidaceae*	*Veillonellaceae, Prevotellaceae*	(17)
103 AS patients and 104 HCs in China	16S rRNA gene sequencing	No significant difference (Observed, Shannon, Simpson, Chao1, ACE and Coverage indices)	Difference (NMDS)	*p_Bacteroidetes, p_Clostridia, p_Betaproteobacteria, f_Bacteroidaceae, g_Megamonas, g_Dorea and g_Blautia, g_Sutterella genera, g_Actinobacteria (f_Coriobacteriaceae), g_Streptococcus, g_Parasutterella, g_Clostridium, g_Bacteroides plebeius*	*p_Bacteroidetes, p_Clostridia, p_Betaproteobacteria, g_Lachnospira, g_Ruminococcus, g_Clostridium_IV, g_Clostridium_XlVb, g_Bacteroides* spp.*, g_uncultured_Christensenellaceae_bacterium, g_Alistipes_shahii, g_Bacteroides_cellulosilyticus*	(18)
127 AS patients and 123 HCs in China	Metagenomic shotgun sequencing	No significant difference	Significant difference (sPLSDA)	*Clostridiales bacterium 1 7 47FAA, Clostridium hatheway, Clostridium bolteae*	*Bifidobacterium adolescentis, Coprococcus comes, Lachnospiraceae bacterium 5 1 63FAA, Roseburia inulinivorans*	(19)

AS, ankylosing spondylitis; HCs, healthy controls; ASns, AS nonsmokers; ASs, AS smokers; HCns, HC nonsmokers; HCss, HC smokers; ASns0, AS nonsmokers at baseline; ASs0, AS smokers at baseline; axAS, axial AS; pAS, peripheral AS. qPCR, quantitative polymerase chain reaction; PCoA, principal co-ordinates analysis; PCA, principal components analysis; NMDS,non-metricMulti-dimensional scaling; sPLSDA, sparse partial least squares discriminant analysis. ND, not done.

**Table 2:** Altered metabolites in AS patients.

Research object	Technology employed	Up-regulated metabolites	Down-regulated metabolites	Ref.
74 AS and 74 HCs (44: plasma and urine, 30: ligament tissue, respectively)	NMR	Plasma: 3-HB, NAG, methionine, acetone, acetoacetate, betaine, glycerolUrine: glycine, hippurate, 2-PYLigament tissue: TG (L2, L3, L5, L6, L7, L8)	Plasma: leucine, valine, alanine, TG (L1, L2, L3, L5, L7, L8), glucose, glutamateUrine: butyrate, creatinine, PAG, glutamateLigament tissue: choline	(20)
24 HCs, 27 CD, 21 axSpA, and 12 CD-axSpA (distal colon tissue and fecal samples)	LC-MS	IAA, I3Ald, indole	omega 3	(21)
33 AS and 33 HCs (serum)	GC-MS	FFAs (C12:0 C16:1 C18:3 C20:4 C20:5 C22:5 C22:6), EFAs (C12:0 C18:3 C22:6)	FFAs (C16:0), EFAs (C16:1 C18:0 C18:1 C18:2 C20:4)	(22)
15 AS and 24 HCs (plasma)	GC-MS and LC-MS	proline, glucose, phosphate, urea, glycerol, phenylalanine, homocysteine	phosphocholines, tryptophan, bipeptide - phenylalanyl-phenylalanine	(23)
10 AS and 10 HCs (serum)	Two-dimensional electrophoresis	pre-Hp	NA	(24)
30 AS, 32 RA, and 30 HCs (serum)	UPLC-TQ-MS	leucine, valine, tryptophan, alanine, creatine, tyrosine, 4-hydroxy- L-proline, arginine, isoleucine, methionine, histidine, lysine	glutamine, glutamate, phenylalanine, serine, proline, γ-aminobutyric acid, creatinine, dimethyl-glycine, taurine, asparagine, acetyl- carnitine, ornithine, citrulline, threonine, glycine, aminobutyric acid	(25)
81 SpA and 86 HCs (serum)	NMR	amino acids, acetate, choline, N-acetyl glycoproteins, Nα-acetyl lysine, creatine/creatinine	low-/very low-density lipoproteins, polyunsaturated lipids	(26)
40 AS, 35 RA, and 34 HCs (fecal samples)	NMR	taurine, methanol, fumarate, tryptophan	butyrate, propionate, methionine, hypoxanthine	(27)

AS, ankylosing spondylitis; HCs, healthy controls; CD, Crohn’s disease; SpA, spondyloarthritis; RA, rheumatoid arthritis. NMR, nuclear magnetic resonance; LC-MS, liquid chromatography-mass spectrometry; GC-MS, gas chromatography-mass spectrometry; UPLC-TQ-MS, ultra-high performance liquid chromatography-triple quadrupole mass spectrometry. 3-HB, 3-hydroxybutyrate; NAG, N-acetyl glycoprotein; TG, triglycerides; PAG, phenylacetylglycine; 2-PY, 2-pyridone-3-carboxamide; L1, CH_3_(CH_2_)_n_; L2, CH_3_CH_2_CH_2_C=; L3, -(CH_2_)_n_-; L5, -CH_2_C=C-; L6, CH_2_CO; L7, C=CCH_2_C=C; L8, -CH=CH-; IAA, indole-3-acetate; I3Ald, indole-3-acetaldehyde; FFA, free fatty acid; EFA, essential fatty acid; pre-Hp, haptoglobin precursor. NA, not available.

**Figure 1:**
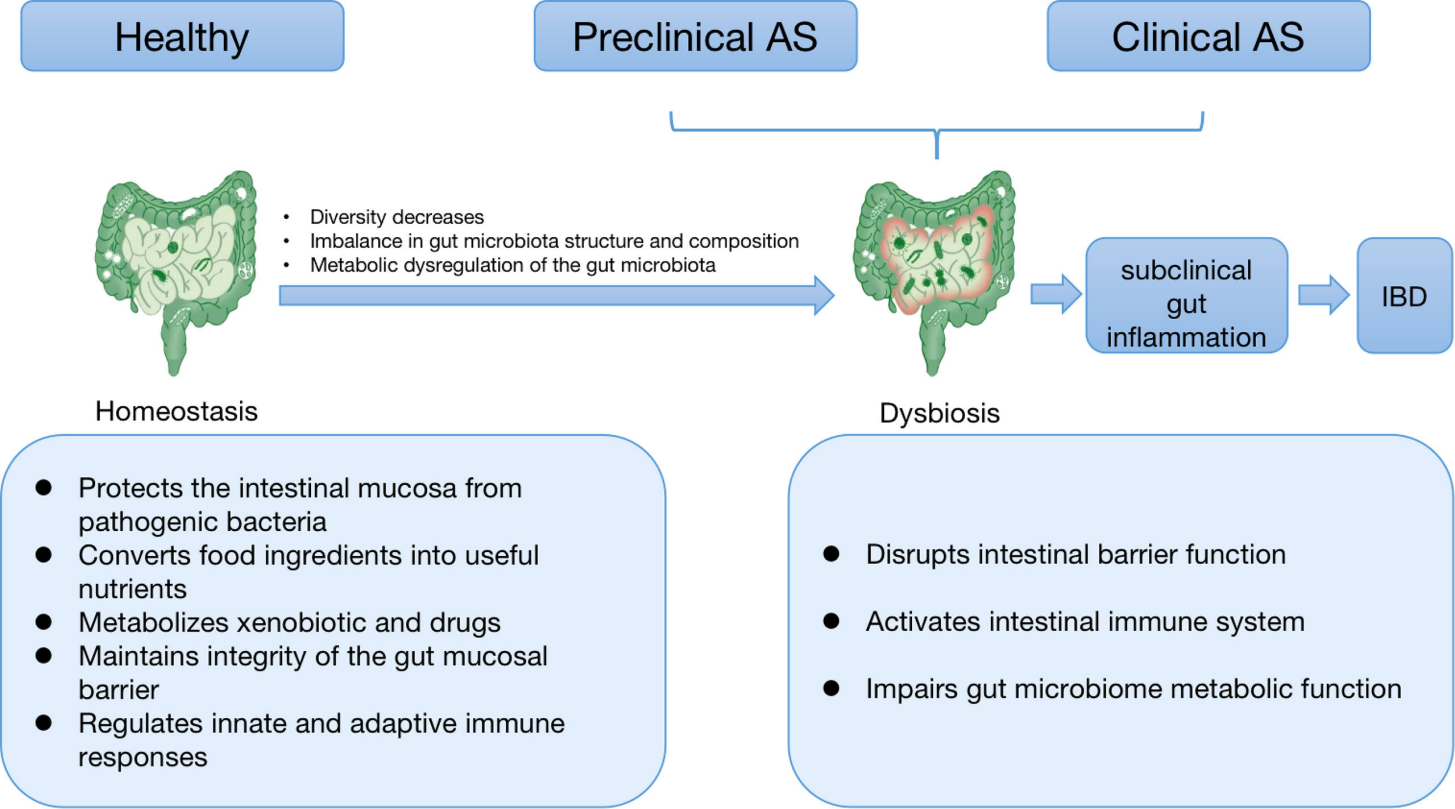
The relationship between intestinal dysbiosis, intestinal inflammation, and AS. Healthy gut microbiota is mainly responsible for human health. Gut microbial homeostasis imparts a specific function in protecting the intestinal mucosa from pathogenic bacteria, converting food components into valuable nutrients, metabolizing xenobiotics and drugs, and maintaining the integrity of the gut mucosal barrier and intestinal immune homeostasis. When species diversity begins to decline, the composition and function of intestinal microbiota change, and metabolism becomes dysregulated, we see a breakdown of the symbiosis, known as dysbiosis. Dysbiosis can disrupt intestinal barrier function, activate the intestinal immune system, and impair the metabolic process of the intestinal flora, thereby leading to subclinical intestinal inflammation. As the inflammation further intensifies, it develops into IBD with clinical manifestations. As is often accompanied by subclinical intestinal inflammation or IBD, some patients have suffered from dysbiosis in preclinical disease. This suggests that AS and IBD may have a common etiology–disturbed gut microbiota. In addition, there is a strong correlation between the severity of gut inflammation and the degree of spine inflammation in AS. Taken together, intestinal inflammation and immune responses in AS may be possible consequences of microbial dysbiosis. AS: ankylosing spondylitis; IBD: inflammatory bowel diseases.

AS patients with subclinical intestinal inflammation, up to 70% of whom 4-16% will progress to IBD (28). The reason for this phenomenon may be that the imbalanced distribution and metabolic disorders of gut microbiota increase the permeability of the gut mucosa, activates the gut immune system, and produces a variety of pro-inflammatory factors, leading to gut inflammation. A cross-sectional study revealed that inflammatory lesions in AS patients with standard and aberrant colonoscopy were independent of bowel symptomatology and treatments used to treat the underlying disease (29). Distinct fecal microbiome signatures associated with gut inflammatory markers such as calprotectin are present in AS patients (15). This suggests that gut inflammatory status is closely associated with the gut microbiota signature of AS patients. In addition, there is an apparent association between the progression of SpA and the severity of chronic intestinal inflammation (30). Some AS patients have suffered from subclinical intestinal inflammation or IBD before the onset of the disease, suggesting dysbiosis in preclinical AS patients. The relationship between intestinal dysbiosis, intestinal inflammation and AS is shown in **Figure 1**.

Many studies have also revealed the critical roles of some bacteria in the pathogenesis of AS. Intestinal *Klebsiella* abundance was closely related to disease activity in AS (31). *Klebsiella* antibodies are closely associated with intestinal inflammation in axial AS patients (32). Wen et al.’s finding demonstrated that *Actinobacteria* are enriched in AS patients and might regulate the ubiquitination of IκB-α. This, in turn, activates NF-κB signaling and promotes the cumulation of pro-inflammatory cytokines in AS patients (16). In addition, fungi or cross-kingdom interactions between fungi and bacteria may contribute to the progression of AS. Elevated antibodies to the fungal cell wall element mannan have been observed in SpA (14). Functional polymorphisms have been identified in genes such as CARD9 and interleukin (IL)23R, which regulate the innate immune response to fungi (33).

Taken together, the gut microbiome and its abnormal metabolism are possible factors in the pathogenesis of AS. Therefore, we should focus on characterizing the species composition and metabolic pathways associated with AS, as well as the mechanisms by which the gut microbiome and its metabolites play a role in disease progression.

## 3 Possible mechanisms of the gut microbiome promoting the development of AS

### 3.1 Intestinal microbiota and HLA-B27

AS development is strongly associated with human leukocyte antigen (HLA)-B27, a major histocompatibility complex (MHC) allele. Several findings back up the concept that intestinal microbiota can increase the risk of AS by interacting with HLA-B27. The HLA-B27 transgenic rats spontaneously developed SpA-like disease, which relies on the gut microbiota to activate the IL-23/IL-17 pathway in the inflamed bowel and joints (34, 35). Interestingly, HLA-B27 transgenic rats cultured under aseptic conditions did not develop apparent inflammatory lesions in the intestine and joints (36). In contrast, normal luminal bacteria predictably and uniformly cause chronic colon, stomach, and joint inflammation in B27 transgenic rats (4), suggesting that the involvement of bacterial flora is necessary for the pathogenesis of HLA-B27.

**Figure 2:**
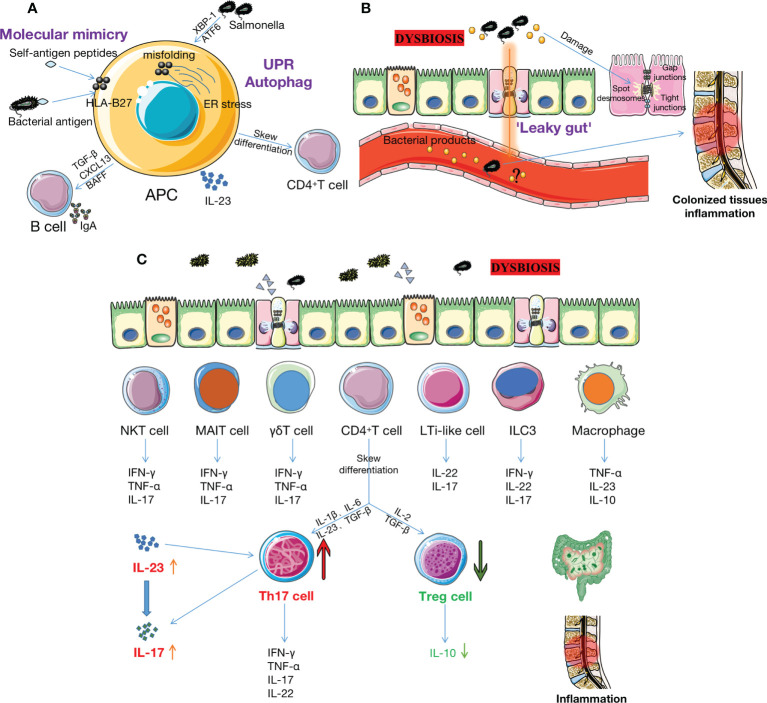
The intestinal microbiome and its metabolites may contribute to AS pathogenesis. **(A)** The interaction of gut microbiota and HLA-B27 may increase the risk of AS through two mechanisms. Firstly, HLA-B27 provides specific peptides, such as certain bacterial antigenic components (e.g.polypeptide structures of Klebsiella and Shigella), to APCs (e.g., CD8+ T or DC cells), resulting in cross-react on molecularly similar self-peptides. Secondly, misfolding of HLA-B27 within ER leads to ER stress, which triggers the UPR or autophagy, activating IL-23/IL-17 axis in the bowel and joints and simultaneously activating B cells to secrete IgA. However, the role of UPR in HLA-B27-driven inflammation has not been demonstrated in humans. **(B)** Dysbiosis leads to increased permeability between IECs, associated with reductions in cathelicidins and mucins, disruption of TJ proteins, impairment of GVBs, etc. Bacteria and bacterial products subsequently enter the bloodstream, inducing inflammation of the colonized tissue. However, it is uncertain whether there is bacterial translocation in AS patients. **(C)** Many immune cells (e.g., Th17, Treg, ILC3, MAIT, γδ T, NKT, LTi-like cells, DCs, macrophage et al.) reside in the intestinal mucosa. Dysbiosis triggers these cells to increase pro-inflammatory cytokines (such as IL-17, IL-23, TNF-α, IFN-γ, IL-7, IL-22, et al.) and decrease anti-inflammatory cytokines (such as IL-10). This activates the IL-23/IL-17 pathway and increases the Th17/Treg ratio, ultimately triggering or exacerbating inflammation in the gut and joints. AS: ankylosing spondylitis; HLA-B27: human leukocyte antigen B27; APCs: antigen-presenting cells; DC: dendritic cell; ER: endoplasmic reticulum; UPR: unfolded protein response; IECs: intestinal epithelial cells; TJ: tight junction; GVB: gut vascular barrier; Th17: T helper 17 cells; Treg: T regulatory cell; ILC3: innate lymphoid cell 3; MAIT: mucosal-associated invariant T cell; NKT: natural killer T cell; IL-17: interleukin 17; TNF-α: tumor necrosis factor-α; IFN-γ: interferon-γ; SCFAs: short chain fatty acids; Trp: tryptophan.

There are two hypotheses regarding the interaction mechanisms between the microbiome and HLA-B27 in AS (**Figure 2A**). One of the hypotheses is the atherogenic peptide theory. This theory suggests that HLA-B27 presents bacteria or autoantigenic peptides to CD8^+^T or B cells, resulting in cross-reactivity with molecularly similar self-peptides (5, 37). Ebringer et al. (38) believed that there was molecular mimicry between HLA-B27 and some bacterial antigen components in the intestinal tract of patients with AS (such AS the polypeptide structure of *Klebsiella* and *Shigella*). *In vitro*, bacterial peptides from AS-enriched species triggered an increase of interferon (IFN)-γ producing cells, mimicking type II collagen. Several independent studies suggested that *Klebsiella pneumonia* may be the most likely etiopathogenetic trigger for AS because of molecular mimicry mechanisms and immune evidence, as well as the molecular similarity between *Klebsiella* and self-antigens (39, 40). Another theory is that HLA-B27 misfolds within the endoplasmic reticulum (ER) lead to ER stress followed by unfolded protein response (UPR) or autophagy, affecting downstream cellular functions that are hypothesized to contribute to the development of AS (41). This phenomenon has been demonstrated in HLA-B27 transgenic rats (42). However, no evidence of UPR transcriptional gene up-regulation was found in intestinal biopsies from AS patients, but autophagy was observed (43). Therefore, whether UPR is involved in human HLA-B27 and gut microbiome-driven inflammation remains unproven. Antoniou et al. found that misfolding of HLA-B27 and UPR cell conditions were associated with enhanced replication in *Salmonella*, which activates XBP-1 and ATF6. Their study provided evidence for the link between the interaction of gut microbiota with HLA-B27 and the pathogenesis of AS (44).

### 3.2 Gut microbiota and intestinal permeability

Several convincing evidences support gut microbiota’s role in maintaining the gastrointestinal tract’s structure and function. For instance, two soluble proteins, P40 and P75, produced by the *Lactobacillus rhamnosus GG* strain prevent cytokine-induced apoptosis of gut epithelial cells through epithelial growth factor receptor (EGFR) and protein kinase C (PKC)-dependent pathways (45). *Bacteroidetes Thetaiotaomicron* expresses the small proline-rich 2A proteins, which are indispensable for maintaining epithelial villous desmosomes structure (46). Intestinal microbiota participates in the development of intestinal microvessels by inducing the transcription factor angiopoietin-3, thereby promoting the structural evolution of intestinal mucosa (47). Lower gut surface area (48), thin villi (48), prolonged cell cycle time (49), and impaired peristalsis (50)were observed in germ-free (GF) mice. Therefore, gut microbiota is essential for maintaining normal gut structure and function. Tight junction (TJ) protein is the key to maintaining the integrity of the epithelial barrier. The abnormal function of TJ proteins leads to IBD even in the presence of normal underlying immunity (51). A mechanism for maintaining TJ is toll-like receptor (TLR)2-mediated signal transduction stimulated by microbial cell wall peptidoglycan. *In vitro* and ex vivo intestinal epithelial cell models suggest TLR2 stimulation promotes PI3K/Akt-mediated cell survival *via* MyD88, effectively preserving TJ-associated barrier assembly against stress-induced injury (52).

Increased permeability between intestinal epithelial cells (IECs) results in the development of AS (**Figure 2B**). Francesco et al. (53) performed ileum biopsy in 50 patients with HLA-B27^+^ AS patients and 20 controls. They found invasive and adherent bacteria, mainly gram-negative bacteria such as *Escherichia coli* and *Prevotella*, were present in the ileum of AS patients, which was related to reduced TJ protein and impaired gut vascular barrier (GVB). Another study observed increased gut permeability in AS patients and their relatives (54), though it is unclear whether pathobiont translocation is involved in AS (55). Endotoxin-induced uveitis associated with AS in mice may provide evidence (56). Epithelial and endothelial leakage often gives zonulin and bacterial products the opportunity to enter the bloodstream, which may activate innate and adaptive immune responses (53). However, we are not sure whether the loss of barrier integrity is a cause or a consequence of dysbiosis.

### 3.3 Gut microbiota and gut mucosa immune imbalance

The gastrointestinal tract (GI) is regarded as the largest immune organ. Many immune cells reside in the intestinal mucosa, such as an innate lymphoid cell (ILC)3, mucosal-associated invariant T (MAIT), γδT, natural killer (NK)T, LTi-like cell, macrophage, neutrophil, dendritic cells (DCs), T cells, B cells et al. These cells play a crucial role in immune homeostasis regulation and suppression of inflammatory damage through innate and adaptive immune responses. Recent studies have shown that the gut microbiome’s composition affects the immune system’s development and modulates immune mediators. Dysbiosis may promote intestinal inflammation by affecting these cells, thereby aggravating the development of AS (57–61) (**Figure 2C**).

#### 3.3.1 Gut microbiota and intestinal mucosal innate immunity

Alterations in the gut microbiome activate Paneth cells (PCs), which release the pro-inflammatory cytokines IL-23 and IL-7, causing lymphoid tissue inducer cells to cluster. Subsequently, ILC3s differentiate and produce IL-17 and IL-22. a4b7-expressing ILC3s migrate in the systemic circulation and accumulate in active inflammatory sites such as peripheral joints and bone marrow-rich peripheral tissues (57).

MAIT cells are a unique type of congenital antibacterial lymphocytes preferentially distributed in the intestine’s lamina propria (LP). Similar to IBD patients, AS patients have lower blood MAIT cell levels than HCs, possibly due to the recruitment of MAIT cells to inflammatory sites such as the gut and joints (62). MAIT cell activation is also associated with disease activity in AS patients. Metabolites produced by bacteria and fungi trigger rapid production of cytokines and chemokines responsible for host immune defense, such as IFN-γ and perforin, as well as production of pro-inflammatory cytokines responsible for the pathogenesis of AS, including IL-17 and tumor necrosis factor (TNF)-α (58, 63). In AS patients, IL-7 produced by intestinal PCs stimulates IL-17 production by MAIT cells (64). In summary, the presence and activation of MAIT cells in the intestines and joints of AS patients further supports the connection between the intestines and joints.

The gut microbiota also stimulates DCs to secrete CXCL13, TGF-β, and B-cell activating proteins, leading to immunoglobulin A (IgA) production and class switching by mucosal plasma cells. Although the underlying mechanism of this function remains unclear, it is speculated that this is mediated by MyD88 signaling in LP and follicular DC cells (65). In addition, reducing IL-10 produced by stimulation to autogenous Bacteroidetes cultures may be a mechanism for the onset and persistence of intestinal inflammation in AS (66).

#### 3.3.2 Gut microbiome and IL-23/IL-17 axis

The IL-23/IL-17 axis is an essential factor in the immunopathogenesis of AS. IL-23, which activated DCs and macrophages mainly secrete, stabilizes the phenotype of T helper 17 (Th17) cells and plays a decisive role in differentiating various subsets of IL-17-secreting cells (67, 68). Th17 cell was initially thought to be the primary source of IL-17. Recent studies found that many other lymphocytes, such as CD8^+^ cytotoxic T, Tc17, γδ T, MAIT, NK, and ILCs cells, could also secrete high levels of IL-17 (69–71). IL-17 promotes T cells activation and stimulates the production of pro-inflammatory cytokines and chemokines by fibroblasts, epithelial cells, endothelial and immune cells such as macrophages (72).

In HLA-B27/β2 microglobulin (β2m) transgenic rats, intestinal inflammation co-occurs with increased IL-23 and IL-17 expression in colon tissue (42). Interactions of cell surface ligands with specific microorganisms induce differentiation of IL-17 and IL-22 secreting cells, thereby programming their pathogenicity (73, 74). Dysbiosis characterized by an increased abundance of *Prevotella* species at mucosal sites may directly or indirectly affect type 17 immune responses through alterations in microbial metabolites or barrier function, thereby leading to a loss of immune tolerance and an increase in pro-inflammatory cytokines (such as IL-23) that trigger AS in susceptible populations. *Prevotella* predominantly activates TLR2, causing antigen-presenting cells to produce Th17-polarizing cytokines, including IL-1 and IL-23. *Prevotella* also stimulates the production of IL-6, IL-8, and CCL20 by epithelial cells, thereby promoting mucosal neutrophil recruitment. This phenomenon leads to the systemic dissemination of bacteria, bacterial products, and inflammatory mediators, thereby affecting systemic disease outcomes (75). All these findings suggest microbial dysbiosis triggers homeostasis changes in intestinal and joint inflammation *via* the IL-23/IL-17 pathway in AS.

#### 3.3.3 Gut microbiome and Th17/Treg

Th17 and regulatory T (Treg) cells are differentiated from CD4^+^T cells. Th17 cells promote tissue inflammation and bone resorption, while Treg cells suppress autoimmunity and maintain immune homeostasis (76). Accumulating Tregs in inflamed joints contributes to the spontaneous resolution and remission process of arthritis in pSpA (77). The proportion of Tregs in peripheral blood monocytes and CD4^+^T cells was significantly reduced, and functional defects of Tregs promote the pathogenesis and progression of AS (78, 79). Several clinic studies observed an increased Th17/Treg ratio in AS (80–82). Reducing the Th17/Treg ratio helps promote the development of new bone formation in AS (83).

The gut microbiota composition modulates Th17/Treg balance in LP and thus may influence gut immunity, tolerance, and susceptibility to AS. Segmented filamentous bacteria (SFB) promotes SAA production in epithelial cells, which activates DCs to produce IL-6 and IL-23, resulting in the generation of Th17 cells (84). In the mouse gut, SFB triggers distorted T cell differentiation through TLR5 (85). Differentiation of Th17 cells also correlates with *Cytophaga-Flavobacterium-Bacteroides* (CFB) bacteria in the gut and is independent of TLRs, IL-21, or IL-23 signaling but requires proper TGF-β activation. The lack of Th17 cell induction in bacteria is accompanied by an increase in Foxp3^+^ Treg in LP (86). In mice, Tregs were most abundant in the colonic mucosa. Active Treg responses dominated by IL-10 production occurred in the gut of AS patients, and the number of Treg cells in LP of GF mice was significantly reduced (87). Still, it could be recovered by supplementation with specific bacteria such as *Bacteroides fragilis*, *Clostridium consortium* (especially Cluster IV and XIVa), and the “altered Scheidler flora” (a mixture of eight identified symbiotic bacteria) (88–90). Polysaccharide A (PSA), the immunomodulatory molecule of *B. fragilis*, promotes the transformation of CD4^+^T cells into Foxp3^+^Treg cells, which actively maintain mucosal tolerance by producing IL-10 during commensal colonization (91). We infer that their metabolic byproducts are sensed by cells of the immune system and affect the balance between pro-inflammatory and anti-inflammatory cells. For instance, *C. Consortium* and *B. Fragilis* have been reported to induce Treg cell differentiation by producing short-chain fatty acids (SCFAs) from dietary carbohydrates (92).

### 3.4 Role of intestinal microbial metabolites in AS

Microbial metabolites, such as SCFAs, tryptophan derivatives, vitamin B, etc., are thought to affect gut barrier function and exert immunomodulatory activities on immune cell subsets and non-immune cells (93). These crucial gut microbial metabolites and their roles in AS are summarized in **Table 3**. Asquit et al. analyzed the host and microbial metabolites of HLA-B27/β2m transgenic rats and found that various microbial and host metabolites were changed before and after the onset of the disease, affecting a variety of metabolisms way. These and other microbially derived biologically active mediators may provide a modality for early diagnosis and treatment of AS (106).

**Table 3:** Intestinal microbiota metabolites and their possible functions in AS.

Intestinal microbiota metabolites	Possible functions
SCFAs	
Acetate	Promote lectin and defensins expression (94). Inhibit HDAC activity and NF-κB activation (95, 96). Improves intestinal epithelial cells defense (97). Stimulate CD4^+^T cells and ILCs to express IL-22 (98). Decrease iNOS, TNF-α, IL-6 and enhance IL-10. Repress ERK1/2 phosphorylation (96). Promote IgA response to microbiota (99). Effects on neutrophils functions (100). Restore macrophage’s response to inflammation (101).
Propionate	Promote lectin and defensins expression (94). Promote cathelicidin expression (102). Enhance MUCs expression (103). Stimulate CD4^+^T cells and ILCs to express IL-22 (98). Block DCs development (104). Effects on neutrophils functions (100). Enhance Tregs (105). Reduce proinflammatory cytokines (e.g., IL-1B, IL-17A, and IFN-γ) and improve colonic inflammation (106). Diminish TNF-α, CINC-2αβ, and NO. Inhibit HDAC activity and NF-κB activation (95). Regulate systemic bone mass (107).
Butyrate	Promote lectin and defensins expression (94). Promote cathelicidin expression (102). Enhance MUCs expression (103). Upregulate TJ proteins (108–110). Induces TJ assembly (111). Stimulate CD4^+^T cells and ILCs to express IL-22 (98). Regulate CD8^+^T cell’s function (112). Effect macrophage function (113). Regulate Th1 and Th17 Cells Differentiation (114). Block DCs development (104). Effects on neutrophils functions (100). Enhance Tregs (105, 115, 116). Decrease iNOS, TNF-α, and IL-6 and enhance IL-10. Repress DNA binding and NF-κB. Repress ERK1/2 phosphorylation (95, 96). Diminish TNF-α, CINC-2αβ, and NO (95). Regulate systemic bone mass (107). Activate the GPR109A receptor (117). Regulate AhR and its target genes (118).
Trp metabolites	
Indole	Activate the AhR pathway (119).
IAA	Reduce the ratios of pro-/anti- inflammatory cytokines. Improved intestinal mucosal barrier function. Increase Tregs and decrease Th17 cells. Activate the AhR pathway. Restore balance among the intestinal microbial community (120).
IAId	Modulate microbial community. Stimulates ILC *via* the AhR to induce synthesis of IL22 (121).
IPA	Activate AhR (122).
AAs	
Arg	Activate innate intestinal immunity (123, 124).
Glu	Modulate microbial community and activate innate immunity in the intestine. Activate NF-κB, PI3K-Akt, and MLCK signaling pathways (124, 125).
Ser	Expanse effector T Cells (126).
Ile/Gln/Trp	Reduce the arthritic grade of intervertebral joints, alter the concentrations of cytokines and modulate the microbial diversity and composition (127).
PAs	
Putrescine	Improve intestinal integrity. Suppress inflammatory cytokine expression (128).
Ppermidine	Suppress inflammatory DCs function. Promoted Treg differentiation (129).
TMAO	Induce M1 macrophage polarization and promote Th1 and Th17 differentiation (130).
other metabolites	
Vitamin B	
Vitamin B2 (riboflavin)	Interactions with MR1 and MAIT TCRs (131).
Vitamin B3 (niacin)	Activate the GPR109A receptor (117). inhibit DCs, and impede the ILC3s proliferation (132). Promote gut barrier function. Promote anti-inflammatory properties in colonic macrophages and dendritic cells (117).
Vitamin B9 (folate)	Increase Tregs (133, 134).
Inosine	Reduce Th1/Th2. Combat autoimmune mediated by Tregs dysfunction (135).
Histamine	Suppress intestinal inflammation (136).
UroA and UAS03	Upregulate epithelial TJ proteins (137).

SCFAs, short chain fatty acids; IAA, indole-3-acetic acid; IAId, indole-3-aldehyde; IPA, indole-3-propionic acid; Arg, arginine; Glu, glutamine; Ser, serine; Ile, isoleucine; Gln, glutamine; PAs, polyamines; TMAO, trimethylamine-n-oxide; UroA, urolithin A; UAS03, UroA potent synthetic analog; HDAC, histone deacetylase; NF-κB, nuclear factor kappa-B; ILCs, innate lymphoid cells; TJ, tight junction; iNOS, inducible nitric oxide synthase; TNF-α, tumor necrosis factor-α; IL-6, interleukin 6; ERK1/2, extracellular signal-regulated kinase 1/2; IgA, immunoglobulin A; MUCs, Mucins; DCs, dendritic cells; Tregs, T regulatory cells; IFN-γ, interferon-γ; CINC-2αβ, cytokine-induced neutrophil chemoattractant-2αβ; TJ, tight junction; Th1, T helper 1 cells; GPR109A, G protein-coupled receptor 109A; AhR, aryl hydrocarbon receptor; Trp, tryptophan; Th17, T helper 17 cells; MR1, MHC (major histocompatibility class) -related protein-1; MAIT, mucosal-associated invariant T; TCRs, T cell receptors; AAs, amino acids; PI3K-Akt, phosphatidylinositol 3 kinase-protein serine-threonine kinase; MLCK, myosin light-chain kinase.

#### 3.4.1 SCFAs

The gut microbiota produces SCFAs, mainly acetate, propionate, and butyrate, which are present in the intestinal mucosa and feces at a molar ratio of about 3:1:1 from food components (138). Phylum bacteroidetes primarily produce acetate and propionate as their predominant metabolic end products, while human colonic butyrate-producing bacteria are *Firmicutes (* 139). SCFAs exert their functions mainly through two distinct pathways: Interaction with G protein-coupled receptors (GPR), GPR41 (FFAR3), GPR43 (FFAR2), and GPR109A (HM74b), and histone deacetylase (HDAC) inhibition. The biological functions of SCFAs in AS can be divided into effects on the intestinal barrier, immune cells, inflammation, and bone metabolism.

##### 3.4.1.1 Impact on the mucosal barrier

SCFAs improve mucosal barrier function. For example, *Bifidobacteria* enhances epithelial-mediated mucosal defenses by producing acetate, thereby inhibiting the transfer of EHEC Shiga toxin from the intestinal lumen to the bloodstream and protecting the host from fatal infections (97). Lectins, defensins, and cathelicidins play important roles in preventing harmful bacterial overgrowth and dysbiosis. *In vitro* studies have shown that SCFAs, especially butyrate, enhance cathelicidin LL-37 expression in colon cells. The interaction of SCFAs with the GPR43 receptor promotes the expression of the C-type lectin RegIIIγ and β-defensins 1, 3, and 4 in intestinal epithelial cells (94, 102). PCs function is altered in AS patients with subclinical intestinal inflammation, resulting in overexpression of PC-associated peptides, particularly human α-defensin 5 (HD-5) (140). *In vitro*, SCFAs can enhance the expression of Mucins (MUCs) in human goblet-like LS174T cells by inhibiting the corresponding histone acetylation and methylation in the promoter regions of HDAC and MUC2, and by AP-1 activation (103). MUCs promote the adhesion of Lactobacillus acidophilus and Bifidobacterium longum to cultured cells and inhibit the adhesion of *Escherichia coli* (141). Studies have shown that the production of MUCs, especially MUC1, in patients with AS is accompanied by an increase in the expression of IL-22 (142). IL-22 has protective effects on intestinal barrier integrity and immunity, and butyrate stimulates CD4^+^ T cells and ILCs to express IL-22 (98). Gene expression analysis of ileal samples in AS patients showed decreased intestinal TJ protein expression, leading to increased intestinal vascular barrier permeability, translocation of bacterial products in the blood, and subsequent intestinal and joint inflammation. This phenomenon is often accompanied by a marked upregulation of zonulin, which antagonizes TJ formation at the epithelial and endothelial levels (53). Butyrate is an essential enhancer of gut barrier function by upregulating TJ proteins (108–110).

##### 3.4.1.2 Impact on immunity

SCFAs play an integral role in regulating energy homeostasis and affecting immune cell function in the host. SCFAs, especially butyrate, directly increased IFN-γ and granzyme B expression by cytotoxic T cells (CTLs) and differentiation of Tc17 cells to a CTL phenotype. This process relies on the strong inhibition of HDACs in CD8^+^ T cells rather than their interaction with specific SCFA-receptors GPR41 and GPR43. In addition, butyrate also increased the production of IFN-γ in CD8^+^ T cells by regulating mTOR activity and cellular metabolism (112). Gut IgA is vital in maintaining intestinal homeostasis and protecting the intestine from inflammation. Wu et al. reported that IgA is regulated by acetate through “metabolite sensing” GPR43. Mechanistically, acetate induces DC to express Aldh1a2, which converts vitamin A to retinoic acid (RA). However, blocking the RA signaling pathway can inhibit the inducible effect of acetate on the production of IgA in B cells (99). Butyrate has an immunomodulatory impact on intestinal macrophage function. Treatment of macrophages with butyrate downregulates LPS-induced pro-inflammatory mediators, including nitric oxide, IL-6, and IL-12. These effects have also been achieved through the inhibition of HDACs (113). After the Na^+^-coupled monocarboxylic acid transporter Slc5a8 transports butyrate and propionate into cells, butyrate and propionate reduce the expression of transcription factors PU.1 and RelB by inhibiting histone deacetylases, thereby blocking bone marrow stem cells differentiate into DCs (104).

The possible mechanism of the effect of SCFAs lies in the regulation of Th17/Treg balance, especially the strong upregulation of CD25^+^Foxp3^+^Treg *in vivo* and *in vitro*, which are mediated by inhibition of HDAC activity, resulting in the increase of anti-Th17 cytokines (such as IL-10 and IL-12) and the downregulation of pro-inflammatory cytokines (such as IL-23 and IL-17) levels in plasma and colonic mucosa (143, 144). Inhibition of T cell HDACs increases p70 S6 kinase acetylation and rS6 phosphorylation, modulating the mTOR pathways required for Th1, Th17, and IL-10^+^T cell generation (145). During Citrobacter rodentium infection, acetate enhances Th1 and Th17 cell induction but reduces the anti-CD3-induced inflammatory response in an IL-10-dependent manner. Butyrate promoted Th1 cell differentiation by increasing IFN-γ and T-bet expression while inhibited Th17 cell differentiation by decreasing IL-17, Rorαan d Rorγt expression. Interestingly, butyrate promoted IL-10 production in both cell conditions, thereby controlling the development of colitis (114). However, Asquith et al. demonstrated that oral propionate administration to HLA-B27/β2m transgenic rats significantly reduced HLA-B27-related intestinal inflammation and pro-inflammatory cytokines. Still, they did not find expression changes in Foxp3^+^ T cells, immunomodulatory cytokines IL-10 or IL-33, and TJ protein zonula occludens 1 (106). Sałkowska et al. investigated the effect of HDAC inhibition on Th17 differentiation. They found that treatment of Jurkat T cells and *in vitro* differentiated Th17 cells with 2 HDAC inhibitors (butyrate and apicidin) resulted in increased RORγT gene expression, which was associated with increased histone H4 acetylation near the proximal promoter of RORγT. In contrast, treatment of naive CD4^+^ cells differentiated into Th17 cells *in vitro* resulted in downregulation of RORγT expression when treated with the same inhibitor. This suggests a complex interaction with other mechanisms that sequentially modulate the expression of RORγT (146). A possible explanation for these studies is that alterations in SCFA content in AS patients lead to impaired Treg function, which results in activation of the IL23-IL17/22 axis and dysregulation of mucosal cells involved in type 3 immunity and regulated by RORγt (e.g., ILC3, invariant natural killer T (iNKT) cells, MAIT cells, and γδ-T cells) (147).

##### 3.4.1.3 Impact on inflammatory and bone metabolism

Dysbiosis is associated with the activation of NLRP3 inflammasome (148). Both autophagy and NLRP3 inflammasome activation can disrupt gut barrier integrity, and the two systems can activate each other (149). SCFAs have been shown to inhibit NLRP3 inflammasome activation in Caco-2 cells and LPS-induced autophagy. Acetate reduces interleukin-1β (IL-1β) production and neutrophil recruitment by inhibiting macrophages from activating inflammasomes and producing reactive oxygen species (ROS) (101). SCFAs also inhibited pro-inflammatory cytokines (e.g., iNOS, TNF-α, IL-1B, IL-17A, IL-6, and IFN-γ) and colonic inflammation while enhancing the expression of anti-inflammatory cytokines, IL-10 (106). Moreover, SCFAs, especially butyrate, diminished the production of CINC-2αβ, TNF-α, and NO by LPS-stimulated neutrophils (95) and have inhibitory effects on phagocytic and killing functions of neutrophils, thus playing an essential role in neutrophil inflammation in the gut of AS (100). NF-κB and ERK signaling pathways are at least partially involved in the anti-inflammatory activity of these SCFAs (96).

In AS, bone metabolism is simultaneously altered by increased bone resorption and juxtaposition of new bone (150). SCFAs are regulators of osteoclast metabolism and bone mass *in vivo*. In a model of experimental murine arthritis, SCFA appears to have an overall beneficial effect on bone - an increase in systemic bone density, a decrease in bone resorption, and a decrease in osteoclasts (107). Propionate and butyrate induce metabolic reprogramming of osteoclasts, enhancing glycolysis at the expense of oxidative phosphorylation, thereby down-regulating essential osteoclast genes such as TRAF6 and NFATc11 (107).

#### 3.4.2 Trp metabolism and AhR

The host-absorbed tryptophan (Trp) is derived entirely from the diet for protein synthesis and other metabolic pathways. Bacteria metabolize Trp to uridine; They also metabolize dietary Trp to indole and other indole derivatives such as indole-3-propionic acid (IPA), indole-3-acetic acid (IAA), and indole-3-aldehyde (IAld) using tryptophanase. The host does not produce indole because tryptophanase is unique to bacteria (151). Indole and its derivatives have different effects on the host, from promoting inflammatory responses to regulating and eliminating inflammation, depending on specific metabolites, experimental models, cells, and receptors (152).

Indole-containing derivatives are absorbed by the host’s intestinal epithelium to indirectly shape the intestinal microbiome and modulate host responses, including barrier and immune functions, through either the aryl hydrocarbon receptor (AhR) or the pregnane X receptor (PXR) (118). This effect was later associated with the induction of Tregs (153). Additionally, the role of AhR in Th17 biology and the control of IL-22 production by T cells was also demonstrated (154, 155). IPA signals directly through epithelial cells within the intestine to maintain and repair the barrier, while IAA and I3Ald signal AhR on ILCs to increase IL-22 expression in the intestinal mucosa (119, 121, 122). Given the protective effect of IL-22 on gut barrier integrity and the expansion of IL-22-producing ILC3 in AS, this may suggest anti-inflammatory feedback. Venkatesh et al. (156) demonstrated that IPA, as a ligand of PXR, promotes intestinal barrier integrity by down-regulating epithelial TNF-α, inducing MDR1, and regulating epithelial junctional complexes. In addition, other Trp-derived metabolites, including Kynurenine, modulate epithelial IL-10R1 activity in an AhR-dependent manner (157). Such action was associated with protection in colitis and promoted epithelial wound healing (122). An assessment of bacterial metagenomics showed that the microbial community in HCs increased Trp synthesis, while the microbial community in axSpA increased Trp metabolism. The authors found that untreated AS patients had significantly lower Trp synthesis than treated AS patients and HCs (19). One study suggested that Trp metabolism to I3Ald and then IAA is one of the mechanisms by which the gut microbiota potentially affects axSpA development (21). Fecal metabolomics in pediatric SpA implicate decreased metabolic diversity, and changes in Trp metabolism are pathogenic factors (158).

#### 3.4.3 Amino acids and their microbial metabolites

Recent convincing results show amino acids (AAs) significantly affect host immune response. For instance, leucine, glutamine, and gamma-aminobutyric acid (GABA) play critical roles in mediating T cell function, including differentiation and activation of T cells, especially Th1 and Th17 cells (159–161). Mechanistically, this effect may largely depend on mTORC1 signaling since the differentiation of Th17 cells and the expression of IL-17 require AA-induced mTORC1 signaling (159). Spermidine spermine and putrescine, the primary polyamines (PAs) in human cells, originate from arginine metabolism. PAs are found in many foods but are also produced by gut microbiota, such as *Bacteroides spp.* and *Fusobacterium spp* (162). The study found that the expression of spermidine was up-regulated in HLA-B27/β2m transgenic rats compared to HCs (106). Liu et al. showed that adding putrescine to weaned piglet diets could reduce the incidence of diarrhea and improve intestinal integrity. Adding exogenous putrescine also decreased the mRNA levels of TNF-α, IL-6, and IL-8, and their upstream regulatory nuclear receptor kappa B p65 subunit in the jejunal mucosa of piglets improved anti-inflammatory function and inhibited inflammatory response (128). Furthermore, spermidine supplementation prevented IFN-α-induced overactivation of DCs and promoted Treg differentiation *in vivo* and *in vitro (* 129).

#### 3.4.4 Trimethylamine and trimethylamine N-oxide

The gut microbiota produces trimethylamine (TMA) from dietary phosphatidylcholine and quaternary amines such as choline, and L-carnitine, which are then converted to trimethylamine-n-oxide (TMAO) by heparin flavin monooxygenase(FMO), especially FMO1 and FMO3. TMAO is associated with systemic inflammatory disease activity (163). TMAO supplementation promotes Th1 and Th17 differentiation and increases the production of proinflammatory cytokines such as IL-17, IL-1β, and IL-6, which are mediated by polarized M1-like macrophages and require NLRP3 inflammasome activation (130). TMAO has been linked to the risk of cardiovascular disease (CVD), such as atherosclerosis, by inhibiting reverse cholesterol transport and increasing the expression of pro-atherosclerotic receptors on the surface of macrophages (164). CVD is a common comorbidity of AS and other SpA diseases, and TMPO may provide new insights into the underlying mechanisms and interventions associated with AS.

#### 3.4.5 Other metabolites of the gut microbiome

B vitamins such as vitamin B2 (riboflavin), vitamin B3 (niacin), and vitamin B9 (folate) are found in milk, eggs, liver, and green leafy vegetables. Nearly all *Bacteroidetes, Fusobacteria*, and *Proteobacteria* possess a Vitamin B biosynthesis pathway (165). The vitamin B metabolites produced by these microorganisms may delay the development of AS by promoting gut barrier function, inducing the proliferation of Treg cells, modulating the production of anti-inflammatory cytokines, and impacting the part of MAIT cells. Niacin is a receptor agonist of GPR109A. Studies have shown that the niacin-GPR109A interaction inhibits the production of IL-23 by colonic DCs, thereby impeding the proliferation of ILC3s in the colonic mucosa (132). There may be abnormal recycling of ILC3 from the gut to the joints, where they act close to the site of joint inflammation (166). Therefore, vitamin B3 produced by microbial metabolism may serve as a biological mediator of ILC3 activation in AS. The inosine (synthesized by the human microbiome, e.g., *Lactobacillus reuteri*) and histamine may inhibit inflammation by activating the adenosine A2A Receptor and histamine type 2 receptor (H2R), respectively. In addition, UroA (a major microbial metabolite derived from polyphenolics of berries and pomegranate fruits) and its potent synthetic analog (UAS03) up-regulate epithelial tight junction proteins by activating AhR- nuclear factor erythroid 2-related factor 2 (Nrf2)-dependent pathways.

## 4 Targeted gut microbiota therapy in preventing or treating AS

**Figure 3:**
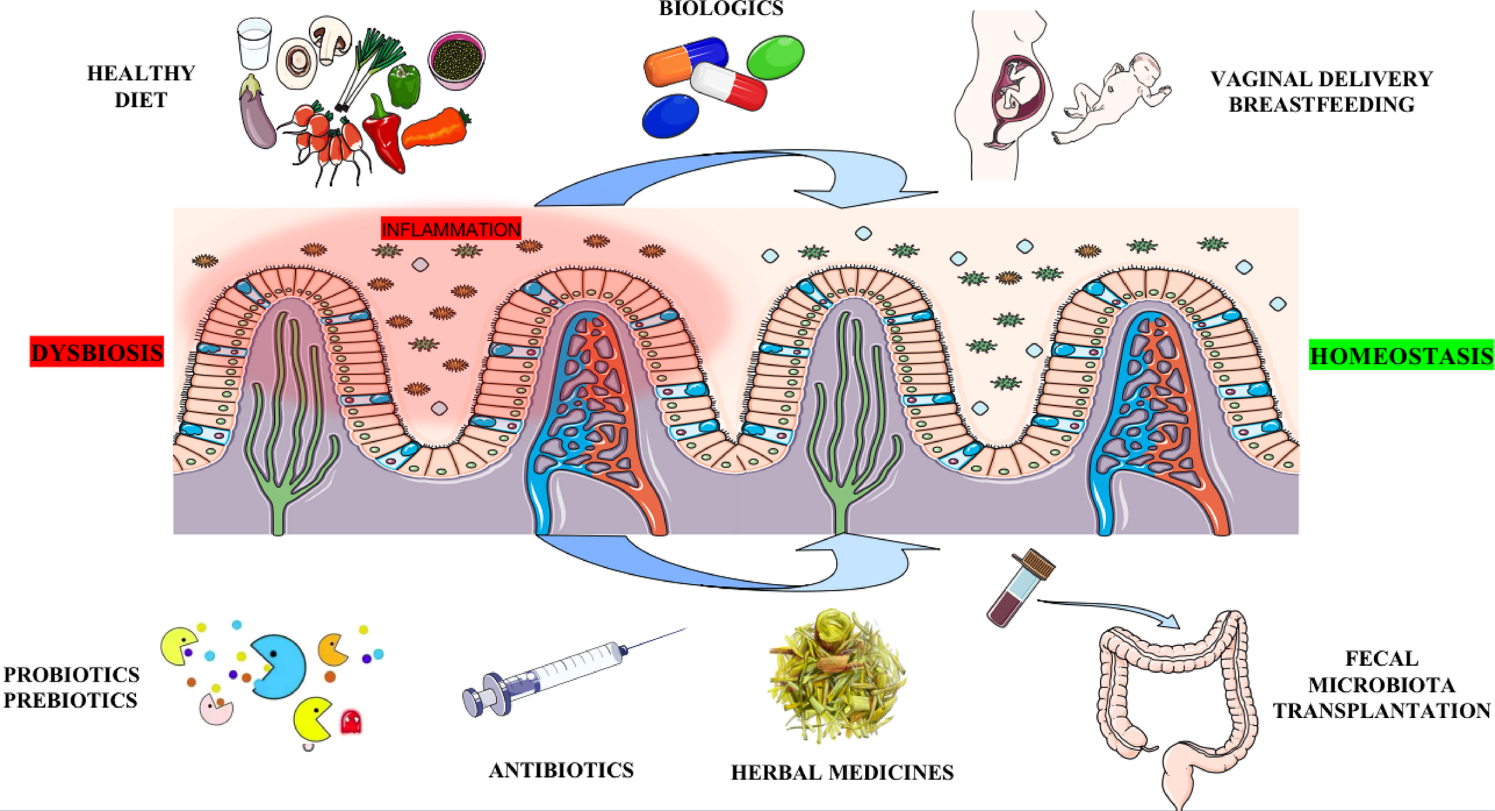
Potential methods for therapeutic intervention targeting the gut microbiota. Antibiotics may eliminate harmful bacteria in the gut. Probiotics, Prebiotics (e.g., SCFAs, Trp metabolites, Vitamin B, etc.), or FMT altering the gut micro-ecosystem serve as a critical strategy by increasing beneficial bacteria. Biologics and Chinese herbal medicine can treat AS by improving gut microecology. Vaginal delivery and breastfeeding participate in the original construction of healthy human gut microbiota. Finally, a healthy diet (such as low starch, high fiber, low fat, low sugar, etc.) fundamentally builds a healthy intestinal microbiota structure, thereby preventing the occurrence of AS. SCFAs: short chain fatty acids; Trp: tryptophan; FMT: fecal microbiota transplant.

The gut microbiota can be influenced by antibiotics, probiotics, prebiotics, medicines, mode of delivery and breastfeeding, diet, and other environmental factors. At present, resetting gut microbial dysbiosis through the methods mentioned above is emerging as a potential approach for the prevention and treatment of AS (**Figure 3**).

### 4.1 Antibiotics

AS is widely suspected as an infectious cause, which is thought to be associated with intestinal microbiota disorders. A variety of antibiotic drugs have been used to treat AS. For example, in a 12-week trial, moxifloxacin was well tolerated, safe, and improved inflammatory symptoms (167). Low dose sulfadiazine sulfapyridine (SSZ), a DMARD and sulfadiazine antibiotic, was influential in seronegative spondyloarthropathies patients with persistently active peripheral arthritis and prevented TNF-α-induced morphologically evident TJ rupture (168, 169). It has been suggested that rifaximin significantly reduced the severity of AS by changing gut microbiome composition with an elevated *Bacteroidete/Firmicutes* ratio and increasing some probiotics selectively, such as *Lactobacillales (* 170). Rifaximin also prevented ileal histological changes, recovered gut barrier function, inhibited the activation of TLR-4/NF-κB signaling, and down-regulated inflammation factors (e.g., IL-17A, IL-6, IL-23, and TNF-α).

However, it has been reported that Extended-Spectrum β-Lactamase (ESBL) production in *Klebsiella pneumoniae* is closely related to resistance to ciprofloxacin (171). Co-resistance with other classes of antibiotics such as tetracycline, chloramphenicol, aminoglycosides, sulfonamides, and fluoroquinolones were also widespread in ESBL-producing strains (172). This reduces the value of AS in the treatment of *Klebsiella pneumonia* infection. In addition, antibiotics may inadvertently eliminate beneficial microbes, leading to an overgrowth of pathogenic and opportunistic pathogens such as *Clostridium difficile* in the gut (173). Since restoring microbiome composition is challenging, antibiotic use should be carefully decided.

### 4.2 Biologics

Etanercept, an anti-TNF-α biologic, plays a crucial role in treating AS, attenuating the incidence and severity of arthritis. In mice, etanercept treatment restored intestinal microbiota composition similar to control mice (174). YIN et al. (19) and Maria et al. (175) found that TNFi treatment substantially restored the disturbed microbiome observed in untreated AS cases, including several critical bacterial species previously associated with AS and other related diseases, compared to HCs. In a study exploring responses to anti-TNF-α therapy, AS patients had a lower microbiota diversity at baseline than HCs at the same level but returned to normal after one month of treatment (12). Potential arthritic bacterial peptides were reduced in AS patients treated with TNFi compared with untreated patients. In addition, etanercept significantly restored TJ protein levels and disruption of ileum tissue, thereby restoring intestinal barrier function. IL-17i, another biologic, caused great changes in the abundance of specific taxa in AS patients, particularly *Clostridiales* and *Candida albicans*. As mentioned above, these data provide insight into biologic therapeutic strategies by modulating the gut microbiome in AS.

### 4.3 Probiotics and prebiotics

Probiotics are a kind of live nonpathogenic microorganisms that improve microbial balance in the gastrointestinal tract (176). The most used probiotic bacterial genera are lactic acid bacteria, mainly from the *Lactobacillus genus*. *Bifidobacterium* is also common, but other genera such as *Enterococcus, Streptococcus*, and *Leuconostoc* are also increasingly used. Also, probiotics may even fall into other fields, such as *Saccharomyces*. The actions of probiotics involve revealing different mechanisms, including production of bactericidal substances, competition with toxins and pathogens for adhesion to the gut epithelium, enhancement of innate immunity and modulation of inflammation through TLR-mediated signaling, promotion of gut epithelial cells survival, barrier function, and protective reactions regulating gut epithelial homeostasis through several signaling pathways (177). On this basis, altering the gut micro-ecosystem could serve as a key target for the prevention or treatment of AS.

*Lactobacillus casei* alleviates joint inflammation in mice with collagen-induced arthritis by reducing Th17 cell production by inhibiting IL-6, IL-17, and IL-23 (178). This effect was also mediated by reducing pro-inflammatory cytokines, inducing anti-inflammatory cytokine IL-10, and inhibiting cyclooxygenase-2 expression. Similar results were reported in an antigen-induced arthritis model study in Lewis rats using the Lactobacillus GG strain (179). Recent studies suggest that butyrate-producing microbes or Akkermansia-type bacteria may have the potential to enhance intestinal barrier function and alleviate inflammation, thereby relieving AS. Considering that SCFAs are largely free of side effects *in vivo*, these and other microbial-derived bioactive mediators may offer new therapeutic modalities for AS (106).

However, in a randomized controlled trial, despite the rationale for the therapy, the probiotic combination did not show a significant benefit compared to a placebo (180). In addition, bacteremia was reported in younger and immunocompromised patients, which was linked to probiotic treatment (181, 182). Therefore, further studies on the bioavailability and biosafety of probiotics in clinical applications are required.

### 4.4 Fecal microbiota transplantation

Fecal microbiota transplantation (FMT) is the transplantation of functional flora in the feces of healthy people into the gastrointestinal tract of patients to rebuild new intestinal flora and treat intestinal and extra-intestinal diseases (183). FMT has been clinically practiced in *Clostridium difficile* infections, which cannot be cured with antibiotics alone (184). In a study of FMT combined with antibiotics in the treatment of UC, it was found that FMT, after antibiotic pretreatment, synergistically promoted the recovery of *Bacteroides* (185). However, more evidence is needed to assess whether FMT can be applied to AS.

Currently, the research on FMT is still in its infancy, and its efficacy and safety still need further consideration because this method is short-lived, requires frequently repeated transplantation, and may transmit pathogenic bacteria, prions, or cytomegalovirus (186). One case reported a young man with UC hospitalized with infectious symptoms, including abdominal cramps, diarrhea, and fever. Not long ago, he received a family FMT (186). In addition to the risk of infection, potential long-term threat to recipients should be noted in the clinical application of FMT. To select donors Carefully assessing donors clinically and not by indicators of gut microbiota composition could theoretically mitigate these risks.

### 4.5 Mode of delivery and breastfeeding

The developing gut microbiota is susceptible to disturbance from external factors, such as parturition and early feeding patterns, maternal nutrition and health, and antibiotic use, especially during the perinatal and early infancy (187–189). Mode of delivery promotes exposure of neonates to specific species: vaginally delivered newborns carry microorganisms of the genera *Bacteroides*, *Bifidobacterium*, *Parabacteroides*, and *Escherichia*. The fecal microbiota of infants born by cesarean section contains *Staphylococcus*(e.g., *Staphylococcus aureus*, *Veillonella*, and *Streptococcus*) and *Enterobacter*. This colonization signature indicates that the bacteria originated from the mother’s oral mucosa, skin, and environment. The effect of delivery mode on intestinal microbiome composition was still detectable at age seven.

Breastfeeding was the most crucial factor regulating the intestinal microbiome after the neonatal period. One study showed that breastfeeding affects the microbiome and can prevent the development of AS (190). Breast milk components provide infants with nutrients and various bioactive compounds that affect immune system stimulation, growth, and regulation, protection from toxins and pathogens, cognitive development, and perhaps most notably, selective colonization and support of a protective microbiome (191–194). Breast milk contains indigestible polysaccharides such as human milk oligosaccharides (HMOs). Recently, studies have shown that only *Bifidobacterium* and *Bacteroidetes* could use HMOs as the sole nutrient and achieved enormous proliferation in the limited number of common intestinal bacteria tested, such as *Clostridium*, *Lactobacillus*, *Escherichia coli*, *Enterococcus isolates*, and *Veillonella (* 195, 196). Interestingly, weaning immediately changes the baby’s microbiome (197).

### 4.6 Diet

Diet is the most direct link between the gastrointestinal tract and external conditions, which affects the composition and function of gut microbiota by affecting their gene expression and metabolites. ZHANG et al. (18) suggested that the gut microbiome alteration in AS was related to dietary factors. Lawrence et al. (198) showed that short-term macronutrient changes alter gut microbiota structure and submerge inter-individual distinction in microbial gene expression, suggesting that gut microbiota can respond rapidly to dietary changes, potentially facilitating the diversity of AS patients’ nutritional lifestyles. Given this scenario, there is growing concern that, as an easily modifiable environmental factor, a diet is an option for the prevention or treatment of AS (199). AS patients themselves consider diet to be more critical than medication (200). Recent studies have shown that the “Western diet” characterized by high starch and fat increases the risk of autoimmune disease by disrupting the gut barrier and affecting the structure and metabolites of microbes (201). Harmful bacteria in the gut depend on dietary starch for growth, so a “low-starch diet” that reduces the intake of “bread, cakes, and potatoes” may be beneficial for AS patients. The ‘low-starch diet’ reduced serum total IgA in HCs and patients, reducing inflammation and symptoms in patients with AS (200). Low fructose diets have also been associated with reductions in markers of inflammation, metabolic syndrome, and oxidative stress (202). Low fiber intake is also associated with an increased incidence of AS (203). Fibers, fermented in the colon, promote bacterial diversity, protect the mucosal barrier, and prompt production of SCFAs, positively maintaining gut microbial homeostasis and preventing inflammation (204–206). High-fiber feeding increases the production of SCFAs, especially acetate and butyrate. Host tolerance to food antigens depends on intestinal mucosal CD103^+^ DCs, which promote differentiation of Treg cells. SCFAs enhance intestinal mucosal immune tolerance and prevent food allergy by increasing retinal dehydrogenase activity in CD103^+^ DCs. This protective effect depends on the vitamin A in the food. This diet promoted IgA production and enhanced T follicular helper and mucosal germinal center responses. Mice lacking the SCFA receptors GPR43 or GPR109A exhibited aggravated food allergy and decreased CD103^+^ DCs. Therefore, a diet that includes fiber and vitamin A is necessary to prevent immune responses to food antigens and to protect the gastrointestinal mucosa (207). Drinking low-fat milk was associated with higher gut microbial diversity, whereas drinking high-fat dairy was associated with lower gut microbial diversity (208). The Mediterranean diet, which involves the intake of high-fiber whole grains, vegetables, and unsaturated fats such as olive oil, nuts, etc., is somewhat similar to an anti-inflammatory diet (209). Adherence to this diet was associated with reductions in inflammatory markers (210, 211). Because of the evidence, this diet seems promising as a possible strategy for addressing AS. In addition, α-Gal, a food allergen, is the leading natural antigen in mammalian red meat and may be involved in the pathogenesis of AS (212). On this account, allergenic foods such as beef, crab, and pork should be excluded from the daily diet of AS patients.

### 4.7 Chinese herbal medicines

As an essential part of complementary and alternative medicines, Chinese herbal medicine has attracted more attention due to its solid curative effect and few side effects. However, the clinical application of Chinese herbal medicines has not been widely recognized due to its complex composition, unclear biological active compounds, and insufficient understanding of their underlying mechanisms. In recent years, herbal medicines have been increasingly used to treat AS patients and play an integral role in modulating gut microbiota during AS management (9). The ingredients of Herbs can modulate the diversity and composition of the gut microbiome (213); Herbal medicines can also affect the metabolism of gut microbiota, such as increasing the production of SCFAs (214); Furthermore, in the interaction of gut microbiota and herbs, herbal compounds can also exert their efficacy by converting into absorbable molecules with potent bioactivity (215). However, this therapeutic approach focuses primarily on relieving pain and delaying disease progression rather than completely controlling or treating AS. Further investigation of herbal medicines may help develop more effective treatments for AS.,,

## 5 Conclusion

There is increasing evidence that gut microbes are involved in the pathogenesis of AS. In-depth characterization of the gut microbiome can be achieved at high throughput and affordable cost through techniques independent of microbial cultures, such as 16s rDNA and metagenomic sequencing. These advanced techniques may provide clues to the intricate role of the gut microbiome in AS pathogenesis. In addition, technological advancements will facilitate the application of microbiome profiling in personalized therapy, providing more effective approaches to treating AS, including narrow-spectrum antibiotics or probiotic therapy, developing healthy eating habits, and introducing whole gut bacteria through FMT. Changes in the gut microbiome are dynamic. Consequently, certain microorganisms can be used as indicators to monitor disease activity and the effectiveness of treatment. In other words, rapid, comprehensive characterization of the gut microbiome may be required for doctors to diagnose and treat patients with AS correctly in the future.

## Author contributions

All authors made a substantial contribution to discussion of the content and to review/editing of the manuscript before submission. Z-YS and DY drafted and wrote the manuscript. S-XZ revised the manuscript. All authors contributed to the article and approved the submitted version.

## Funding

We acknowledge the National Natural Science Foundation of China (Grant No. 82001740) for invaluable funding.

## Conflict of interests

The authors declare that the research was conducted in the absence of any commercial or financial relationships that could be construed as a potential conflict of interest.

## Publisher’s note

All claims expressed in this article are solely those of the authors and do not necessarily represent those of their affiliated organizations, or those of the publisher, the editors and the reviewers. Any product that may be evaluated in this article, or claim that may be made by its manufacturer, is not guaranteed or endorsed by the publisher.
